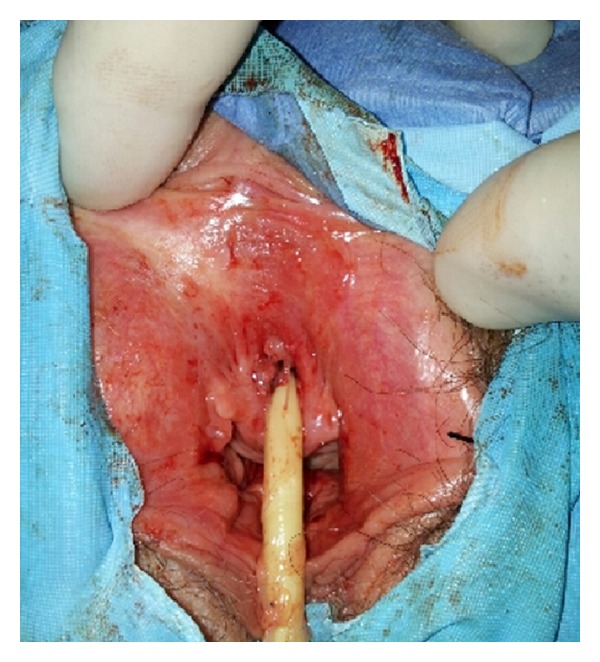# Spontaneous Postmenopausal Urethral Prolapse Treated Surgically and Successfully

**DOI:** 10.1155/2014/695471

**Published:** 2014-07-21

**Authors:** I. Klein, Y. Dekel, A. Stein

**Affiliations:** Department of Urology, Carmel Medical Center and Bruce Rappaport Faculty of Medicine, Technion, Michal 7 Street, 34362 Haifa, Israel

## Abstract

Urethral prolapse (UP) is a circular complete eversion of the distal urethral mucosa through the external meatus. It is a rare condition seen mostly in African-American premenarcheal girls. Both a medical and a surgical approach to this condition have been described. We present a case of a spontaneous urethral prolapse in a 60-year-old postmenopausal Caucasian woman, who failed medical management and underwent successful surgical management. The patient is asymptomatic 18 months following the procedure.

## 1. Introduction

Urethral prolapsed (UP) is a rare and benign condition, with an incidence of 1 : 3000, especially among prepubertal, primarily African-American girls (~80%) [1]. The etiology remains unknown. A popular hypothesis suggests the lack of estrogen or poor attachments between the layers of smooth muscle around the urethra [2, 3]. In recent years there have been a few reports of UP as a result of injecting urethral bulking agents for stress urinary incontinence [4] and spontaneous prolapse [5].

UP presents as a circular and complete eversion of the urethral mucosa at the external meatus. On physical examination, this circumferential lesion of mucosa may look as a red/pink/purple ring, sometimes infected or ulcerated, around what appears to be the external meatus.

The spontaneous postmenopausal cases tend to be symptomatic, with vaginal bleeding being the most common presenting symptom, although dysuria, hematuria, urinary frequency, urgency, and nocturia may also be present.

There is still controversy regarding treatment of this rare entity ranging from conservative treatment options (i.e., topical antibiotics, estrogen creams, and “sitz baths”) as first-line [6] to different procedures, from simple manual reduction to complete surgical excision [7].

We present a case of a large spontaneous UP managed successfully by a surgical approach.

## 2. Case Report

A 60-year-old Caucasian female presented with a few weeks of dysuria, frequency, urgency, and hematuria without fever or chills. She also reported a long standing “lump” in her vulva. Nitrofurantoin 100 mg TID combined with Phenazopyridine TID did not improve her symptoms, nor did topical ointments or sitz baths. Vaginal examination revealed a uterine and cervical prolapse of mild degree and a reddish partly necrotic lump 3 cm in diameter around the area of the urethral meatus.

Transvaginal ultrasound demonstrated a normal sized uterus with thin layered mucosa without masses or pelvic fluid.

Laboratory evaluation showed complete blood count and blood chemistry were within normal limits and urinalysis showed leukocyturia and erythrocyturia with no bacterial growth on culture.

## 3. Surgery

**Figure 1:**
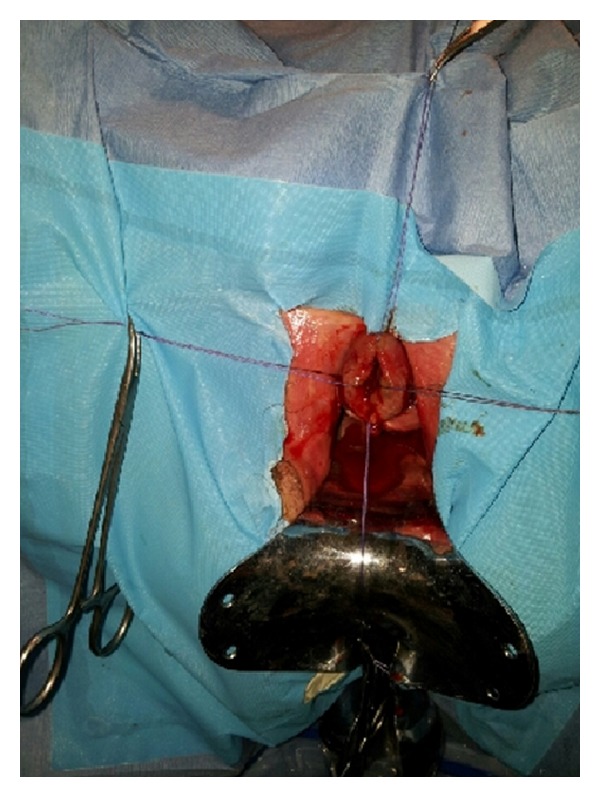


The mass was carefully dissected using four-quadrant excisional technique [8] (Figure 1). The patient was placed in the lithotomy position. Holding sutures were placed in the four quadrants of the prolapsing urethra. The edematous tissue was cut quadrant by quadrant, and in each one a mucosal to mucosal anastomosis was performed, before proceeding to the next quadrant. Traction on the holding sutures allowed good visualization of the proximal mucosa, and prevented its retraction towards the bladder. Once the tissue had been excised and repaired, cystoscopy was performed.

Gross pathological examination of the specimen showed irregular fragments of tissue in a total dimension of 3.5∗2∗1.5 cm with areas of bleeding.

Histopathology showed fragments of urethra with severe inflammation ulceration and cavernous hemangioma.

The urinary catheter was withdrawn at postoperative day 5. Within 2 weeks there was marked clinical improvement with reduction of the periurethral swelling; no mass was identified (Figure 2). On follow-up visits 18 months past the procedure the patient has no bleeding nor urinary symptomatology of any kind with no periurethral bulging on examination.

## 4. Discussion

UP is a rare encounter among adult female patients. In choosing this patient's management we had to bear in mind the severity of her symptoms, a high recurrence rate after pure medical treatment, and the differential diagnosis that includes, among others, urethral or vaginal malignancy, condyloma, and rhabdomyosarcoma.

In this case we were faced with a highly symptomatic otherwise healthy patient, who failed prior medical treatment and was in need of prompt relief of her symptoms. According to the literature, surgical excision still remains the most definitive therapy. There are several surgical approaches described, especially among children, like tying the prolapsed tissue on a catheter [9]. The most frequent complications described are urethral stricture and postoperative urinary retention [1]. We preferred the “four-quadrant excisional technique” [8] that to our opinion achieves a more complete resection and is more anatomic. The patient is symptom-free 18 months past the procedure.

## Figures and Tables

**Figure 2:**